# Evaluation of EEG Headset Mounting for Brain-Computer Interface-Based Stroke Rehabilitation by Patients, Therapists, and Relatives

**DOI:** 10.3389/fnhum.2020.00013

**Published:** 2020-02-14

**Authors:** Mads Jochumsen, Hendrik Knoche, Preben Kidmose, Troels Wesenberg Kjær, Birthe Irene Dinesen

**Affiliations:** ^1^Department of Health Science and Technology, Aalborg University, Aalborg, Denmark; ^2^Department of Architecture, Design and Media Technology, Aalborg University, Aalborg, Denmark; ^3^Department of Engineering – Bioelectrical Instrumentation and Signal Processing, Aarhus University, Aarhus, Denmark; ^4^Department of Neurology, Zealand University Hospital, Roskilde, Denmark; ^5^Laboratory of Welfare Technologies, Telehealth and Telerehabilitation, Department of Health Science and Technology, Aalborg University, Aalborg, Denmark

**Keywords:** brain-computer interface, stroke rehabilitation, usability, EEG headset, technology transfer

## Abstract

Brain-computer interfaces (BCIs) have successfully been used for motor recovery training in stroke patients. However, the setup of BCI systems is complex and may be divided into (1) mounting the headset and (2) calibration of the BCI. One of the major problems is mounting the headset for recording brain activity in a stroke rehabilitation context, and usability testing of this is limited. In this study, the aim was to compare the translational aspects of mounting five different commercially available headsets from a user perspective and investigate the design considerations associated with technology transfer to rehabilitation clinics and home use. No EEG signals were recorded, so the effectiveness of the systems have not been evaluated. Three out of five headsets covered the motor cortex which is needed to pick up movement intentions of attempted movements. The other two were as control and reference for potential design considerations. As primary stakeholders, nine stroke patients, eight therapists and two relatives participated; the stroke patients mounted the headsets themselves. The setup time was recorded, and participants filled in questionnaires related to comfort, aesthetics, setup complexity, overall satisfaction, and general design considerations. The patients had difficulties in mounting all headsets except for a headband with a dry electrode located on the forehead (control). The therapists and relatives were able to mount all headsets. The fastest headset to mount was the headband, and the most preferred headsets were the headband and a behind-ear headset (control). The most preferred headset that covered the motor cortex used water-based electrodes. The patients reported that it was important that they could mount the headset themselves for them to use it every day at home. These results have implications for design considerations for the development of BCI systems to be used in rehabilitation clinics and in the patient’s home.

## Introduction

Brain-computer interfaces (BCIs) provide a means for users to control external devices using only their voluntarily produced brain activity (Wolpaw et al., 2002). Control signals can be extracted from electroencephalographic signals (EEG) which are then processed to enhance the signal-to-noise ratio. From the cleaned signals, features are extracted and classified into different classes corresponding to different device commands. Examples of this could be up/down/left/right movement of a cursor or robotic arm or initiation of a rehabilitation robot (Millán et al., 2010). Over the past years, BCIs have been used for stroke rehabilitation (Cervera et al., 2018) by decoding the patient’s intention to move the affected body part and then provide sensory feedback through a rehabilitation robot (Ramos-Murguialday et al., 2013), electrical stimulation (Biasiucci et al., 2018), or virtual reality/visual feedback (Pichiorri et al., 2015). The beneficial effect of BCI-based rehabilitation training on motor recovery in stroke has been reported consistently (Cervera et al., 2018).

A BCI-based rehabilitation training session could proceed like this; a therapist mounts the headset and prepares the electrodes to reduce electrode impedance to obtain a higher quality EEG signal. In this paper we define mounting as preparing and fitting the cap. Then the patient is asked to perform several movements, 30–50 movements, to calibrate the classifier in the BCI system, and then the actual training starts where the BCI decodes the movement intention generated from the motor cortex and triggers the external device that provides feedback. Such BCI training has a beneficial effect on motor recovery (Cervera et al., 2018), but there are a number of pitfalls when using BCI technology outside the laboratory without a BCI expert/engineer (Leeb et al., 2013; Käthner et al., 2017). These include the complexity of the EEG headset setup (in terms of effort), robustness and performance of the BCI system, and system calibration before each use. Usability (effectiveness, efficiency, and satisfaction; International Organization for Standardization, 1998) has not been explored in great detail within the BCI literature (Kübler et al., 2014), but it has to be considered for the technology to be adopted in clinical practice (Signal et al., 2018). As outlined, the usability testing within BCI literature is limited especially within the area of stroke rehabilitation where only a single study has been identified where the authors followed a user-centered design of a BCI system and assessed the patients’ motivation satisfaction and workload (Morone et al., 2015). In general, the studies that have been conducted on stroke rehabilitation using BCIs, the therapists/experimenters have been mounting the cap/headset. But a potential future scenario could be that the patients would mount the headset and set up the BCI themselves and train without the need of a therapist being present. The therapist can then spend more time on other types of training and patients, or the patients could be training in their own home. Many of the caps currently commercially available may be difficult to mount by oneself since the electrodes must cover the motor cortex to record the electrical activity associated with attempted movements, and only a few comparisons between headsets or headset usability have been made (Ekandem et al., 2012; Mayaud et al., 2013; Das et al., 2014; Hairston et al., 2014; Nijboer et al., 2015; Halford et al., 2016; Izdebski et al., 2016; Pinegger et al., 2016; Käthner et al., 2017; Zander et al., 2017; Radüntz and Meffert, 2019). These studies relied on different metrics but often report on comfort and setup time. Other aspects covered by them include the unobtrusiveness (Das et al., 2014) and design elements such as adaptability of head sizes, external trigger integration, cap fit, and variance in scalp electrode locations (Hairston et al., 2014; Izdebski et al., 2016). Moreover, the satisfaction using questionnaires and system preferences are reported as well (Hairston et al., 2014; Nijboer et al., 2015; Pinegger et al., 2016; Käthner et al., 2017). Another aspect to consider is the aesthetics of the headset, which has been reported to be important (Nijboer et al., 2014). Most evaluations of headsets and electrode types used healthy subjects, but a few included veterans and severely motor impaired patients (Halford et al., 2016; Käthner et al., 2017), caregivers (Käthner et al., 2017). Only in two studies, participants – healthy volunteers – mounted the headset themselves (Zander et al., 2017; Radüntz and Meffert, 2019). The studies that have investigated EEG headset usability have primarily focused on communication using P300 and EEG recordings, and not on stroke rehabilitation and self-mounting. Therefore, there is a need for an EEG headset evaluation for stroke rehabilitation and neurorehabilitation in general which are important applications for BCI. This study explores an end-user’s point of view the feasibility of using different types of headsets with different electrode types for stroke rehabilitation including self-mounting that would enable patients to use a BCI in their home. They will be mounted by the different stakeholders involved in the rehabilitation; patients, therapists and relatives. The core metrics include the setup time of the headsets, comfort, preferences, and implementation considerations in clinical practice. Three headsets will have electrode positions that cover the motor cortex while the remaining two headsets are placed on the forehead and around the ear. The latter two headset are included as a control and are used as a reference for potential design considerations although they are not configured for stroke rehabilitation, i.e. do not record signals from the motor cortex.

## Materials and Methods

### Participants

Nine motor impaired patients participated in this study (Three females and six males; age: 50–76 years). They were recruited from a rehabilitation clinic in the Aalborg Municipality (Traeningsenheden Aalborg Kommune). The inclusion criteria for the patients were that they could understand the protocol and be able to participate for 2 h; varying degrees of motor impairment were accepted. They all gave written informed consent prior to participation. The local ethical committee of Region North Jutland approved all procedures (N-20130081). Moreover, eight therapists working with stroke patients were recruited from the municipality rehabilitation centre (Traeningsenhed Nord, Vodskov, *n* = 4) and a regional rehabilitation clinic (Brønderslev Neurocenter, *n* = 4), and two relatives were included as well. The details of each participant are presented in Table 1. To provide an indication of the patients’ level of upper limb functionality, they answered on how they washed their hair, they could answer; (1) with both hands, (2) with one hand, and (3) I cannot wash my hair.

**TABLE 1:** General information about the participants.

Patient	Injury	Affected side (extremities)	Months since injury	Hair wash
P1	Ischemic stroke	Left	41	1 hand
P2	Ischemic stroke	Right	17	1 hand
P3	Ischemic stroke	Both	2	2 hands
P4	Ischemic stroke	Right	4	2 hands
P5	Sclerosis	Left	288	2 hands
P6	Ischemic stroke	Right	10	1 hand
P7	Hemorrhagic stroke	Right	92	Cannot wash the hair
P8	Ischemic stroke	Right	58	1 hand
P9	Cerebral palsy	Both	At birth	1 hand

**Therapist**	**Occupation**	**Seniority (years)**	**Affiliation**	

T1	Occupational ther.	10	Regional rehabilitation clinic	
T2	Occupational ther.	15	Regional rehabilitation clinic	
T3	Physiotherapist	8	Regional rehabilitation clinic	
T4	Physiotherapist	9	Regional rehabilitation clinic	
T5	Physiotherapist	5	Municipality rehabilitation clinic	
T6	Student	0	Municipality rehabilitation clinic	
T7	Physiotherapist	5	Municipality rehabilitation clinic	
T8	Occupational ther.	2	Municipality rehabilitation clinic	

**Relative**	**Relation**	**Living together**		

R1	Spouse of P8	Yes		
R2	Spouse of P7	No		

*Age: 50–76 years. Three females and six males participated.*

### EEG Headsets

The following paragraphs provide a description of the headsets. The reader should keep in mind that our study tested the headsets for an application in stroke rehabilitation for which the headsets had not been developed, where patients, therapists and relatives were mounting the headsets. Five headsets were chosen to cover a wide price range and different electrode types; dry, gel-based, and water-based electrodes. Three of the headsets covered F3, Fz, F4, C3, Cz, C4, P3, Pz, and P4. These channels have been used previously to record one of the control signals (movement-related cortical potential) for BCIs used in stroke rehabilitation (Jochumsen et al., 2015a, c). The other two headsets were located on the forehead and around the ear, so they did not cover the motor cortex; these headsets were considered as a control to the other headsets.

#### Ultracortex “Mark IV”: OpenBCI

**FIGURE 1:**
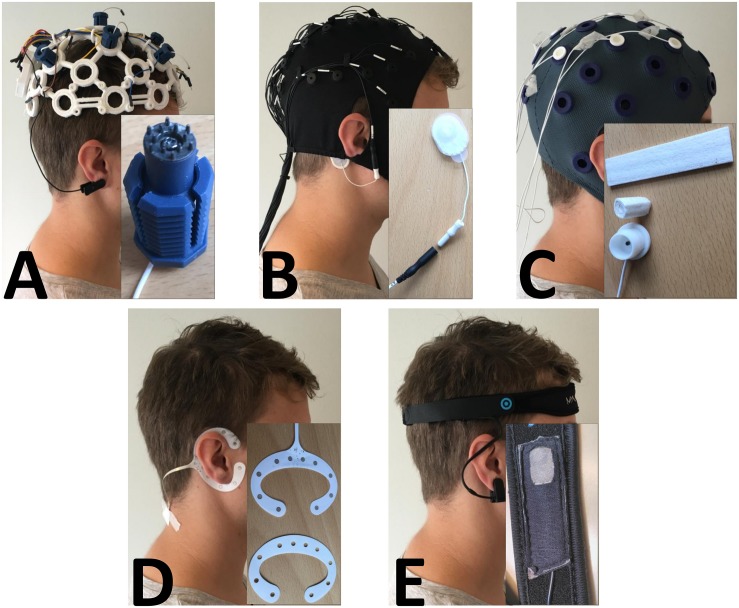
Picture of each headset with a close-up of the sensor. **(A)** OpenBCI with the dry pin electrode. **(B)** Quick-Cap with the connector to the reference electrode. **(C)** Water-based electrode with the felt “insert” and the electrode housing. **(D)** cEEGrid with a picture of the double-sided adhesive tape that needed to be fitted precisely to the electrode. **E:** MyndBand with a picture of the dry electrode.

The headset from OpenBCI (Figure 1A) has the possibility to be configured in various ways. There were 35 electrode locations, and up to 16 channels could be sampled. The headset could be 3-D printed in three different sizes (small, medium, and large), and the electrodes could be screwed into place in the cap at the electrode locations. The system was tested with dry pin electrodes. In this study, nine electrodes were mounted in the following positions: F3, Fz, F4, C3, Cz, C4, P3, Pz, and P4. An ear clip electrode was used as the reference, and any of the nine electrodes could be used as a ground electrode. The headset is fitted such that all electrodes have contact with the scalp. The OpenBCI had to be mounted such that all electrodes had contact with the scalp, and the ear clip was placed on the right earlobe.

#### Quick-Cap: Compumedics Neuroscan

The Quick-Cap headset/cap from Compumedics Neuroscan (Figure 1B) has 34 fixed electrodes and a ground electrode (AFz) placed according to the International 10-20 system. There were six leads where electrodes could be attached (reference channels and recording of eye activity). The cap was made of elastic breathable Lycra material, and it came in different sizes (small, medium, and large). To prepare the electrodes, gel was used to fill up the small neoprene cups so there was contact between the electrodes and the scalp. In this study, the following electrode positions were used: AFz, F3, Fz, F4, C3, Cz, C4, P3, Pz, and P4. The reference electrode was an Ambu^®^ Neurline 720 electrode that was connected to one of the reference leads. The reference was placed on the mastoid bone behind the right ear. The setup for the Quick-Cap included the following: (1) filling a syringe with gel, (2) connecting the reference electrode with the connector on the lead, and (3) placing the cap on the head, tighten the chinstrap, place the reference electrode on the mastoid bone (behind the ear) and fill the neoprene cups with gel.

#### Water-Based Electrodes: TMSi

The Water-based electrodes from TMSi (Figure 1C) consist of a felt “insert” that is rolled tight and placed in a plastic water-electrode housing. Then the electrode was soaked in water and inserted in the cap which had 33 fixed electrode positions. The cap was made of elastic soft fabric. In this study, the following electrode positions were used: F3, Fz, F4, C3, Cz, C4, P3, Pz, and P4. For recordings, a common average reference would be applied, and the signals would be grounded to a wrist band. The setup for the Water-based electrodes also had three steps: (1) roll the felt “inserts” and place them in the housing, (2) soak the electrodes in water and place them in the cap, and (3) mount the cap and tighten the chinstrap.

#### cEEGrid: TMSi

The cEEGrid electrode from TMSi (Figure 1D) has 10 fixed electrodes on a flex printed, multi-channel sensor array, which was placed behind the ear. It was fixed with double-sided adhesive tape that needed to be changed each time it was used. The adhesive tape that came with the cEEGrid had been fitted to the electrode, and there was a small hole for each of the 10 electrode sites. To prepare it, the adhesive tape had to be placed on the electrode, so each hole on the adhesive tape fitted each hole on the electrode. Then a drop of gel was placed in each hole and the other side of the adhesive tape was removed. The electrode was placed behind the ear of the less-affected side and fitted such that there was no hair between the skin and the electrode. For recordings, a common average reference would be applied, and the signals would be grounded to a wrist band. The setup of the cEEGrid consisted of: (1) filling a syringe with gel, (2) remove one side of the double-sided adhesive tape, position the tape on the electrode, fill the holes with a drop of gel, remove the other side of the adhesive tape, and (3) place the electrode behind the ear without getting hair between the electrode and skin. The cEEGrid was placed behind the ear on the patient’s less affected side.

### MyndBand: MyndPlay

The MyndBand from MyndPlay (Figure 1E) consists of a dry electrode that was mounted on a neoprene headband and placed on the forehead. The back of the electrode was fixed with Velcro on the headband, so it could be moved laterally and medially. The headband was adjusted using Velcro. The dry electrode was referenced using an ear clip electrode. There was no information about grounding of the electrode. The MyndBand was placed on the forehead, it had to be relatively tight, and the ear clip was placed on the earlobe.

### Experimental Procedure

The order of the headsets mounted was randomized on each participant. Each headset had to be worn 15 min by the patients since some headsets had been reported to become uncomfortable over time. Initially, the participants were instructed on how to set up the headsets and the instructions were given and showed immediately before the setup of each headset. The experimenter showed the setup of the headset and the steps to perform; this lasted approximately 5 min. The participants could ask questions if they were in doubt about the procedure. The patients could see themselves in a mirror while setting up. The therapists and relatives mounted the headsets on healthy volunteers. No signals were recorded in this study, and no impedance measures were used, the 1st author (10 years of EEG and BCI experience) qualitatively judged if the headset was mounted correctly. The check included if the electrodes/headset was in the right position, if there was hair between the electrode and the scalp for the cEEGrid, and if there was contact between the electrodes and the scalp. The total setup time was measured as well as the individual tasks (e.g. three measurement for the Quick-Cap). After each headset had been worn the participants filled in a questionnaire with respect to the specific headset.

### Questionnaire

The questionnaires were presented in the participant’s first language (Danish). They consisted of six questions for the patients and four questions for the therapists and relatives for each headset. The patients had to evaluate the comfort and aesthetics on a 10-point Likert scale from 1 (very poor) to 10 (very good). All participants had to evaluate the difficulty of setting up the headset, the anticipated amount of help need, and the overall rating of the headset on the same 10-point Likert scale. All the participants had to rate on a 5-point Likert scale if they thought that other patients/therapists/relatives would be able to set up the headset. After all headsets had been tested, the participants had to rate the headsets by overall preference from 1 to 5 where 1 was the best. Lastly, some general questions were asked. The patients were asked if the aesthetics of the headset and if hair wash after each use was a problem; these were rated on a 5-point Likert scale. All participants were asked what the maximal setup time should be if they were to use the headset every day (5 min intervals). The patients and therapists were asked if it was important that the patients could set up the headset themselves; this was rated on a 5-point Likert scale.

### Statistics

A 1-way repeated measures analysis of variance (ANOVA) test with “headset” as factor was used to investigate if there was a difference between the total setup time for the four different headsets. This was only tested for the therapists since six patients could not complete the setup of all headsets. Significant test statistics were followed up with a Bonferroni *post hoc* test. The estimated effect size was reported as well. The answers for the questionnaires and the overall headset preference were analyzed using two Friedman’s tests for patients and therapists, respectively. Significant tests were followed up with Wilcoxon tests using a Bonferroni correction. Statistical significance was assumed when *P* < 0.05. Kendall’s concordance coefficient (W) was reported as well. The relatives were not included in the statistical analysis due to the sample size.

## Results

For the OpenBCI headset, every subject, except one, complained about pain, and said that it was too uncomfortable to wear. The last subject had not tightened the chinstrap enough to allow adequate contact between the electrodes and the scalp. Therefore, this headset was left out from further analysis since the headset had to be worn for 15 min.

### Setup Time

**FIGURE 2:**
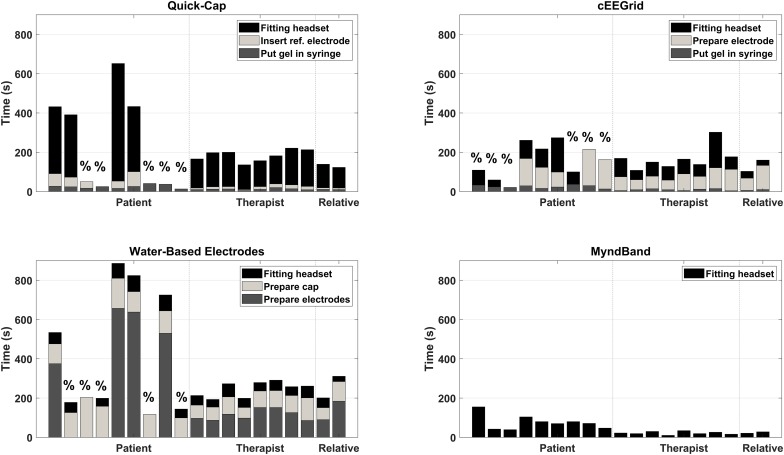
Overview of the setup time for each headset for every participant. The “%” indicates the patients that could not complete the entire setup of the headset. The dashed vertical lines indicate the separation between patients, therapists and relatives.

**FIGURE 3:**
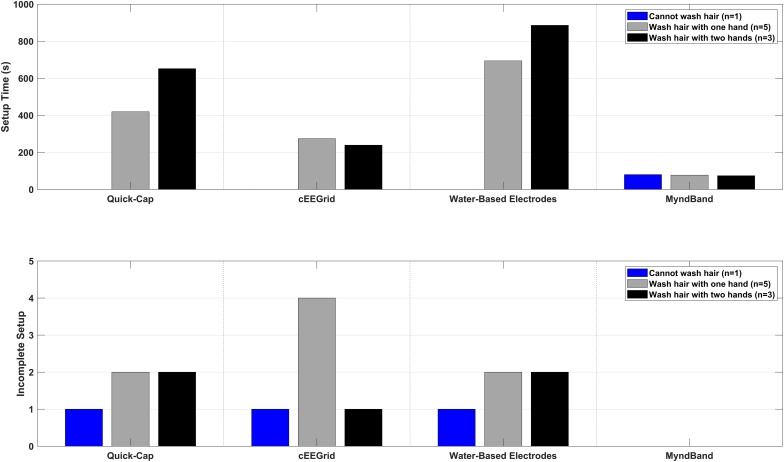
Overview of the setup time and number of incomplete setups in three sub-groups of the patients. The sub-groups were: (1) patients who cannot wash the hair, (2) patients who can wash hair with one hand, and (3) patients who can wash hair with both hands.

The results of the setup time are shown in Figures 2, 3. Most of the patients (5–6 out of 9) could not complete the entire setup for the Quick-Cap, cEEGrid, and Water-based electrodes. For the Quick-Cap, the difficulty was to insert the reference electrode in the connector on the cap and filling the electrodes with gel when the cap was placed. The difficulties with cEEGrid was to prepare the electrode by aligning the holes in the self-adhesive tape with the electrode sites on the electrode. Moreover, those that could mount the electrode did not do it correctly primarily because of hair getting in the way. The major limiting factor of the Water-based electrodes was electrode preparation where the felt needed to be rolled tightly to fit into the electrode cup/holder. All patients could mount the MyndBand. The therapists could mount all of the headsets, but there was a significant difference between the setup time [*F*_(3, 21)_ = 65.7; *P* < 0.001; η^2^ = 0.90]. The setup time were different for all headsets except the Quick-Cap and cEEGrid. The fastest setup of the headsets covering the motor cortex was the Quick-Cap, but both the Quick-Cap and the headset with Water-based electrodes took less than 5 minto mount for the therapists and relatives. As expected, the fastest setup was for the MyndBand for patients, therapists and relatives which took around 0.5–2 min. In Figure 3, the setup time and number of patients that could not perform the setup are presented. The patients are divided into three categories based on the question if they could wash their hair (Table 1). For the patient who was unable to wash the hair only the MyndBand could be mounted by the patient. There was no clear trend of a faster setup time for patients who could use both hands for hair wash compared to those who only could use one hand.

### Headset Evaluation

The results of the questionnaire are summarized in Table 2.

**TABLE 2:** Results of the headset evaluations for patients, therapists, and relatives.

	Quick-Cap	cEEGrid	Water-based	MyndBand
**Patients**				
Comfort	[6, 8, 9.5]	[9.5, 10, 10]	[4.5, 7, 8.5]	[9, 10, 10]
Aesthetics	[5.5, 7, 9]	[9.5, 10, 10]	[4.5, 8, 9.5]	[9.5, 10, 10]
Setup difficulty	[1, 3, 4.5]	[2, 5, 7.5]	[1, 3, 3.5]	[8, 10, 10]
Question	[1, 2, 3.5]	[3, 4, 5]	[1, 3, 4]	[3.5, 5, 5]
Need for help	[1, 1, 4.5]	[1.5, 3, 8.5]	[1, 3, 8]	[9, 10, 10]
Satisfaction	[4.5, 5, 7]	[7, 9, 9.5]	[2.5, 5, 7.5]	[9, 10, 10]
Preference	[2.5, 4, 4]	[1.5, 2, 2.5]	[3, 3, 3.5]	[1, 1, 1.5]
**Therapists**				
Setup difficulty	[6, 7, 9.5]	[3.75, 7, 8]	[5, 8.5, 10]	[10, 10, 10]
Question	[4, 4.5, 5]	[4, 5, 5]	[3.5, 5, 5]	[5, 5, 5]
Need for help	[6.5, 9, 9.75]	[8.25, 9.5, 10]	[6, 10, 10]	[9.25, 10, 10]
Satisfaction	[7.25, 8, 9.75]	[5.75, 8, 8.75]	[5.5, 9, 10]	[8, 10, 10]
Preference	[2, 3, 3.75]	[3, 3.5, 4]	[2, 2.5, 3.75]	[1, 1, 1]
**Relatives**				
Setup difficulty	[9, 10]	[10, 10]	[9, 10]	[10, 10]
Question	[4, 4]	[5, 5]	[4, 5]	[5, 5]
Need for help	[10, 10]	[9, 10]	[10, 10]	[10, 10]
Satisfaction	[9, 9]	[8, 9]	[9, 10]	[10, 10]
Preference	[4, 4]	[2, 2]	[3, 3]	[1, 1]

*The results for the patients and therapists are presented as percentiles [25, median, 75], and the individual results for the relatives are presented. Everything except “Question” and “Preference” is rated from 1 (worst) to 10 (best). The “Question” was: “I think other patients/therapists/relatives will learn to set up the headset quickly” and it was rated from 1 (strongly disagree) to 5 (strongly agree). The headset preference was rated from 1 (best) to 4 (worst).*

The patients rated in general the headsets to be comfortable, but there was a significant difference between them [χ^2^_(__3__)_ = 15.2; *P* = 0.002; *W* = 0.56] with the MyndBand and cEEGrid electrode being more comfortable than the Water-based electrodes.

The patients rated the aesthetics relatively high, but there were some differences in their rating of the individual headsets. There was a difference between the headsets [χ^2^_(__3__)_ = 12.7; *P* = 0.005; *W* = 0.47], the MyndBand and cEEGrid were rated higher than the Quick-Cap when using the non-adjusted *P*-value (none of the comparisons differed significantly after applying the Bonferroni correction).

In general, the patients rated the headset setup to be difficult for all headsets except for the MyndBand, but the therapists and the relatives found it easy to mount all of the headsets. There was a difference between the patient’s perceived setup difficulty of the headsets [χ^2^_(__3__)_ = 16.3; *P* = 0.001; *W* = 0.60]. The patients reported the MyndBand to be significantly easier to mount than the headsets covering the motor cortex. For the therapists [χ^2^_(__3__)_ = 9.1; *P* = 0.03; *W* = 0.38] there was a difference in the perceived difficulty as well, the MyndBand was easier to mount than the cEEGrid, but not with respect to the Quick-Cap and Water-based electrodes.

The participants were asked if they thought other patients/therapists/relatives would be able to learn the setup quickly. It varied for the headsets in the patient group with the headsets covering the motor cortex receiving lower scores than the MyndBand and cEEGrid. There was a significant difference between headsets [χ^2^_(__3__)_ = 18.4; *P* < 0.001; *W* = 0.68] with the MyndBand being quicker to learn to mount compared to the Quick-Cap and the Water-based electrodes. The therapists’ and relatives’ high scores indicated that they expected all headset setups would be quick to learn. There was no difference between the expected need of help for setting up the headsets [χ^2^_(__3__)_ = 4.6; *P* = 0.21; *W* = 0.19].

The patients rated that they would require much help for all headsets except the MyndBand. There was a difference between headsets [χ^2^_(__3__)_ = 13.4; *P* = 0.004; *W* = 0.50] with the MyndBand being different from the Quick-Cap. The therapists did not expect to require much help, but there was a difference between headsets [χ^2^_(__3__)_ = 8.2; *P* = 0.04; *W* = 0.34]. The MyndBand required significantly less help than the Quick-Cap when using the non-adjusted *P*-value (there were no significant comparisons when applying Bonferroni correction).

The median of the patients’ rating of the overall headset satisfaction was 5 for the headsets covering the motor cortex while it was rated higher for the MyndBand and the cEEGrid. There was a difference between headsets [χ^2^_(__3__)_ = 17.7; *P* = 0.001; *W* = 0.65] with the MyndBand receiving higher scores than the Quick-Cap and the Water-based electrodes. The therapists and relatives gave high ratings for all systems, and there was no difference between the overall satisfaction with the headsets [χ^2^_(__3__)_ = 6.3; *P* = 0.10; *W* = 0.26].

In general, the MyndBand was preferred across the three participant groups. There was a significant difference between the headset preference among the patients [χ^2^_(__3__)_ = 12.6; *P* = 0.006; *W* = 0.47] with the MyndBand being preferred (rated number 1) over the Water-based electrodes (rated number 3) and the Quick-Cap (rated number 4). The headset preference was also different for the therapists [χ^2^_(__3__)_ = 13.5; *P* = 0.004; *W* = 0.56] where the MyndBand was preferred over the Quick-Cap and cEEGrid.

### General Design/Implementation Considerations

**FIGURE 4:**
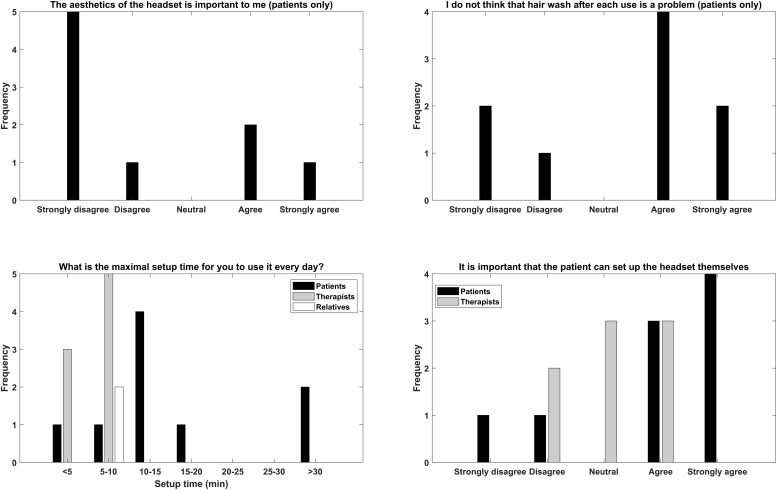
Overview of the replies of the general question the participants were asked regarding aesthetics, hair wash after use, maximal setup time, and self-mounting.

The answers of the questionnaires related to general design and implementation considerations are presented in Figure 4. For the aesthetics and washing the hair, there were mixed answers with the majority stating that the aesthetics were not important and hair wash was not a problem. A general comment from the patients was that if the BCI training works then the aesthetics was not a problem, however, since no EEG signals were recorded this issue was unaddressed. For the maximal setup time there was consensus that the setup should take less than 15 min. It was important for the patients to able to set up the headset themselves especially those that did not have relatives living with them.

## Discussion

In this study, it was investigated how different headsets fit into supervised or unsupervised stroke rehabilitation in clinical practice or the patient’s home seen from an end-user’s perspective. The MyndBand was the fastest to mount, the only one to be mounted by all patients, and it was generally preferred by all participants. It should be noted that the MyndBand does not record electrical from the motor cortex which is needed for an application for stroke rehabilitation. The therapists and relatives were able to mount all headsets within 5 min, which was within the self-reported limits of the maximum setup time if they were to mount the system every day. The dry pin electrodes were not included in the analysis as the participants found them too uncomfortable to wear. The Water-based headset was the most preferred headset that covered the motor cortex among the patients, therapists and relatives.

### Setup and User Evaluation

The fastest headset to mount was the MyndBand, as expected, due to the simplicity of the setup and the fact that only a single dry electrode needs to be placed on the forehead. Similar setup times were shown in other studies for a similar simple setup (Ekandem et al., 2012). However, this electrode position is not relevant for detecting movement intentions from the motor cortex, and it took a considerably longer time (up to 10 times as long) to mount the other headsets, and six out of nine patients were unable to mount the headsets by themselves. This is primarily due to their motor impairments, and that it was difficult to fill the electrode housings with gel in the Quick-Cap using the syringe, placing the double-sided adhesive tape on the cEEGrid electrode and rolling the felt inserts for the Water-based electrodes, healthy users also have difficulties in setting up gel-based headsets (Radüntz and Meffert, 2019). This suggests that the current headsets based on wet electrodes that cover the motor cortex are not suited for self-mounting by stroke patients. A potential design consideration could be e.g. to roll and place the felt inserts in the water-electrode house, which is where many patients struggled, such that the patients only should apply water to the electrodes and place the headset on their head. In general, the therapists and relatives had no problems in mounting the different headsets; this indicates that all headsets covering the motor cortex can be used if the therapists are mounting the headsets and there is a relative or caregiver in the patient’s home that can help with mounting the headset. The patients rated the comfort quite high, which is consistent with previous findings (Hairston et al., 2014; Nijboer et al., 2015; Oliveira et al., 2016), except for the dry pin electrodes in the OpenBCI headset, which was too uncomfortable to wear. The reported results related to the comfort of dry electrodes have been mixed, some studies found them not uncomfortable (Guger et al., 2012; Halford et al., 2016), others uncomfortable, especially when the headset had been worn for some time (30–90 min have been reported) (Hairston et al., 2014; Oliveira et al., 2016; Käthner et al., 2017; Radüntz and Meffert, 2019). It should be noted that the patients in our study only wore the headsets for 15 min, which is likely to be a bit shorter than a training session would last, and that the comfort is gradually reduced over time if electrodes are exerting pressure or if headsets are bulky (Mayaud et al., 2013; Hairston et al., 2014; Zander et al., 2017). Although wet electrodes were comfortable, several of the patients rated the need for a hair wash after use as a problem, thus we believe this is an important design constraint to consider. A solution could be more comfortable dry electrodes or using just a single self-adhesive wet electrode (Jochumsen et al., 2015c). The aesthetics of the headsets were rated high, and mixed results were obtained about the importance of the aesthetics of the headsets. End-users and caretakers found the aesthetics of the BCI system important (Nijboer et al., 2014). However, these end-users are from a severely motor impaired patient group where they will be using a BCI permanently throughout the day for communication and control. For BCI-based stroke rehabilitation training, the training is only done in a limited time period each day. Some of the participants stated that they did not care about aesthetics if the BCI training was beneficial for their recovery, and that they would just use it at home or in the rehabilitation clinic. Likewise, it has been reported in a study with healthy participants that they would not accept less comfort for better aesthetics (Radüntz and Meffert, 2019). The patients found the setup of headsets covering the motor cortex both difficult and requiring help. This indicates that if the patients are supposed to mount the BCI themselves, then the headset should be easy to use like the MyndBand. However, if the patients have assistance for mounting the headsets several systems can be used. According to the therapists, the patients do not necessarily need to be able to mount the headset since they would be able to help the patients with that, however, it is a matter of resources which differ in different countries.

### Limitations and Future Studies

The interpretation of the results should consider the limited sample of patients, therapists and relatives. A larger sample, including participants from different countries, would provide more accurate and generalizable results. The experimental period was short, so it is not known if the results would be different after the participants have tried it several times. It is likely that there would be a learning effect of mounting the headset, but it is expected that this effect was reduced by training the participants in setting up the headsets and performing the various steps (e.g. placing self-adhesive tape on the cEEGrid electrode) immediately before the actual test.

The aim of this study was to put the stakeholders in the rehabilitation in focus, and to highlight a number of concerns that are important to address for self-fitting and potentially home use. However, an important limitation of this study was that the effectiveness of BCI performance was not tested (no signals were recorded), which is considered to be part of the usability testing of a BCI system (Kübler et al., 2014). The limitation of the effectiveness is an important point since the headsets in this study cover different electrode positions (spatial coverage on the scalp) and number of electrodes. The MyndBand and cEEGrid were rated the highest as expected since the electrode positions are in positions without hair. These two headsets are only considered as a control for the other headsets since the recording sites are not directly overlying the motor cortex where the control signals for the BCI used in stroke rehabilitation normally are recorded (Jochumsen et al., 2015b, c; Frolov et al., 2017). However, design considerations from e.g. the MyndBand can be used to design a headset with a single electrode covering the motor cortex, which has been shown to be sufficient to record MR (Jochumsen et al., 2015c). Thus, it may be a trade-off between headset simplicity and eventually BCI performance. Future studies should compare signal quality and movement intention detection between the headsets and in other contexts such as the patient’s home.

## Conclusion

In general, patients would prefer to be able to mount the headset themselves, but they needed help to mount the headsets that covered the motor cortex. The therapists and relatives were able to mount all of the headsets. According to the patients, a BCI-based rehabilitation training system for home use, the ease of use of the headset should be comparable to the MyndBand. However, several headsets that cover the motor cortex can be used for a BCI training system in a rehabilitation clinic context in which the patients have access to help during setup from a usability testing point of view (disregarding the effectiveness), the therapists and relatives were satisfied with all of the headsets. Our results provide input for the design process of headsets for BCI-based rehabilitation systems that must be transferred from research laboratories to rehabilitation clinics or the patient’s home.

## Data Availability Statement

The datasets generated for this study are available on request to the corresponding author.

## Ethics Statement

The studies involving human participants were reviewed and approved by The North Denmark Region Committee on Health Research Ethics. The patients/participants provided their written informed consent to participate in this study.

## Author Contributions

MJ collected and analyzed the data. HK, PK, TK, and BD helped in interpreting the results and critically revised the manuscript. MJ drafted the manuscript. All authors were approved the final version and involved in the design of the study.

## Conflict of Interest

The authors declare that the research was conducted in the absence of any commercial or financial relationships that could be construed as a potential conflict of interest.
